# Personal

**Published:** 1916-10

**Authors:** 


### PERSONAL

Announcement of removal, travel, and other matters of interest are requested. Please report errors in the listing of any physician in the State and other directories, that we may co-operate with the State Society in securing a correct list.

---

Dr. Lester I. Levyn of Buffalo announces the removal of his laboratory of Roentgenology to 444 Franklin Street. Hours 9-5, by appointment.

---

Dr. Charles William Hennington of Rochester, announces the removal of his office and residence to 633 Park Avenue.

---

Misses Bessie M. Scanlon, Alice Holman and Margaret Murday of Buffalo, have sailed with a Red Cross Unit under the direction of Dr. Earl B. Downer of Lansing, Mich. It will proceed to Petrograd via Christiana and will be assigned to duty in the Caucasus.

---

Frank Leroy Purdy, A. M., M. D., University of Buffalo 1895, who has been recuperating his health at the Jackson Health Resort, has opened an office at 554 Elmwood Avenue, Buffalo.

---

Dr. Nahum Kavinoky of Buffalo, has returned from visiting the surgical clinics at Rochester, Minn., Chicago and Cleveland.

---

Dr. Herman M. Biggs, State Health Commissioner, was operated upon in September by the Mayos, for appendicitis.

---

Dr. Milton E. Bork of Buffalo has been appointed Acting Superintendent of the Good Samaritan Dispensary.

---

Dr. J. P. Barr of Buffalo spent the latter part of August and first of September in Atlantic City.

---

Dr. Lucius J. Waterman of Buffalo returned September 22 from a motor trip down the state.

---

Dr. W. H. Coe, of Auburn, has removed from 82 East Genesee Street to his newly furnished offices and residence, 165 Genesee Street.

---

Dr. R. F. Johnson, of Auburn, has removed from 12 James Street to 161 Genesee Street.

---

Dr. Lawrence Sisson, of Auburn, has recently opened an office for general practice at 162 Genesee Street.

